# Fibro Adenomata of the Breast

**Published:** 1908-01-11

**Authors:** 


### Points in Surgery.

### FIBRO-ADENOMATA OF THE BREAST.

The title fibro-adenoma of the breast comprises the great majority of the simple tumours which affect this organ, because it includes every gradation of new growth from pure fibroma at one extreme, to pure adenoma at the other. Pure fibroma itself is exceedingly rare; in fact, it may be said that a fibrous tissue new growth of the breast, uncomplicated by the presence of glandular tissue, is almost unknown; and, at the other end of the scale, pure adenoma is excessively uncommon, for it stands to reason that if such a tumour exists, it must be difficult to differentiate from normal breast tissue, even under the microscope. The difference is said to lie in the irregular arrangement of the glandular tissue in the adenoma; but it is obvious that even this is a question on which expert opinion may vary considerably.

An overwhelmingly large proportion of simple tumours of the breast consist of new formations of tissue which is partly fibrous and partly adenomatous. Some authorities reserve the term "fibro-adenoma" for those in which the connective tissue element preponderates, while those in which the adenomatous tissue is in excess they call "adeno-fibromata." This subtle distinction seems unnecessary; and in this article all such tumours are classed under the name of "adeno-fibroma." The tumours consist typically of a localised overgrowth of breast tissue, which consists of acini and ducts as in an ordinary resting breast. The acini are supported by a matrix of fibrous tissue, and more or less surrounded by a fibrous capsule. The phrase "more or less surrounded" is used because, although the capsule is complete in the great majority of them, this is not invariably the case.

Mr. Bowlby, in his "Surgical Pathology," subdivides adeno-fibromata of the breast into three classes, according to the microscopical appearances of the adenomatous tissue. He recognises (1) Adeno-fibroma acinosum. In this form the glandular tissue resembles in appearance and in the proportion of its elements normal resting breast. (2) Adeno-fibroma tubulare. Here the acini are almost completely absent, and all that is seen is a number of cross-sections of duct-like structures lined by columnar epithelium. (3) Adeno-fibroma intracanaliculare. In this last variety, which may be regarded as an advanced form of the second, the duct-like structures are invaded by ingrowths of the fibrous matrix, so that their lumen becomes highly irregular, and in places only represented by ramifying clefts or slits. In any given tumour, especially if it has grown to a considerable size, all three types may be found existing side by side, if sections are cut from different parts of it.

In most cases the matrix consists of ordinary fibrous tissue; but in some, to quote Mr. Bowlby, "it is rather cellular in type and very commonly shows a tendency to mucoid degeneration." Although these tumours are typically innocent in nature, the transition to malignancy is one of easy stages, and the diagnosis depends entirely on the microscopical characters of the interstitial tissue. Where this shows retrogressive changes, and is taking on the appearance of embryonic connective tissue cells, the tumour must be regarded as an adeno-fibro-sarcoma, and such tumours afford us a very good example of neoplasms originally innocent becoming malignant secondarily. For there can no longer be any reason to doubt that it is impossible to draw a hard and fast line between innocent and malignant neoplasms. Certain it is that it cannot be done on clinical grounds, and although histological examination affords us a far more certain means of diagnosing between the two, there are many cases in which even expert microscopists confess themselves unable to give an opinion. Many other examples might be quoted of tumours arising in different parts of the body which are certainly innocent originally, and which as certainly become malignant later. To take a single example, the frequency with which a papilloma of the tongue or of the lip becomes epitheliomatous is a case in point, and the lesson which we should learn from such cases is this—that every so-called innocent tumour should be regarded as potentially malignant, and a danger to the life of its possessor. The very presence of an innocent tumour should be taken as denoting a lack of equilibrium in the normal tissue tension, which allows one set of cells to grow at the expense of others; and who shall say that that is an innocent phenomenon? What we have to do is to make a thorough and systematic microscopical examination of all so-called innocent tumours that are removed, and it may be that if we can catch even one of these growths actually in the act of becoming malignant, the secret of that mystery which now baffles the medical profession, the ætiology of malignant disease, may be disclosed to us.

But what, it may be asked, is the practical outcome of these reflections? That all these adeno-fibromata of the breast should be examined microscopically when removed, however obvious it may appear on clinical grounds that they are innocent.

And the clinical evidence is all in favour of innocence. The tumour is generally single, and is rounded or oval. It can be easily palpated with the flat of the hand, and in a typical case moves with remarkable freedom in the breast tissue. Of course, if it is not completely encapsulated, its mobility is much diminished, and this is also the case if it is situated deeply in the substance of the mamma. It is firm to the touch, or may give a sensation of elasticity, grows slowly, and is generally painless. The characteristic signs of carcinoma mammæ are wanting; *i.e.*, the axillary glands are not enlarged the nipple is not retracted, and the skin over the tumour does not dimple. An adenofibroma should be removed as soon as diagnosed. If it is superficial this should be done by means of an incision radiating from the nipple; but, if it lies deep, it can be done by making a crescentic incision round the lower border of the breast and enucleating the tumour from the deep aspect. The resulting scar will hardly be visible.

